# Infarction in the territory of the anterior cerebral artery: clinical study of 51 patients

**DOI:** 10.1186/1471-2377-9-30

**Published:** 2009-07-09

**Authors:** Adrià Arboix, Luis García-Eroles, Núria Sellarés, Agnès Raga, Montserrat Oliveres, Joan Massons

**Affiliations:** 1 Unit of Cerebrovascular Diseases, Service of Neurology, Hospital Universitari del Sagrat Cor, Universitat de Barcelona, Barcelona, Spain; 2 CIBER de Enfermedades Respiratorias (CB06/06), Instituto Carlos III, Madrid, Spain; 3 Unit of Organization, Planning and Information Systems, Consorci Sanitari del Maresme, Barcelona, Spain

## Abstract

**Background:**

Little is known about clinical features and prognosis of patients with ischaemic stroke caused by infarction in the territory of the anterior cerebral artery (ACA). This single centre, retrospective study was conducted with the following objectives: a) to describe the clinical characteristics and short-term outcome of stroke patients with ACA infarction as compared with that of patients with ischaemic stroke due to middle cerebral artery (MCA) and posterior cerebral artery (PCA) infarctions, and b) to identify predictors of ACA stroke.

**Methods:**

Fifty-one patients with ACA stroke were included in the "Sagrat Cor Hospital of Barcelona Stroke Registry" during a period of 19 years (1986–2004). Data from stroke patients are entered in the stroke registry following a standardized protocol with 161 items regarding demographics, risk factors, clinical features, laboratory and neuroimaging data, complications and outcome. The characteristics of these 51 patients with ACA stroke were compared with those of the 1355 patients with MCA infarctions and 232 patients with PCA infarctions included in the registry.

**Results:**

Infarctions of the ACA accounted for 1.3% of all cases of stroke (*n *= 3808) and 1.8% of cerebral infarctions (*n *= 2704). Stroke subtypes included cardioembolic infarction in 45.1% of patients, atherothrombotic infarction in 29.4%, lacunar infarct in 11.8%, infarct of unknown cause in 11.8% and infarction of unusual aetiology in 2%. In-hospital mortality was 7.8% (*n *= 4). Only 5 (9.8%) patients were symptom-free at hospital discharge. Speech disturbances (odds ratio [OR] = 0.48) and altered consciousness (OR = 0.31) were independent variables of ACA stroke in comparison with MCA infarction, whereas limb weakness (OR = 9.11), cardioembolism as stroke mechanism (OR = 2.49) and sensory deficit (OR = 0.35) were independent variables associated with ACA stroke in comparison with PCA infarction.

**Conclusion:**

Cardioembolism is the main cause of brain infarction in the territory of the ACA. Several clinical features are more frequent in stroke patients with ACA infarction than in patients with ischaemic stroke due to infarction in the MCA and PCA territories.

## Background

Cerebral infarcts in the territory of the anterior cerebral artery (ACA) are infrequent and yet few studies have specifically assessed the clinical characteristics of stroke patients with ACA infarction [1]. So far, single case reports and small series of ACA stroke have been reported and, in most cases, patients with ACA infarction are included in group of hemispheric cerebral infarction as a whole, independently of the different vascular topography of lesions [1-3]. In this respect, some aspects of the natural history of ACA infarction, such as aetiology, clinical features and prognosis have not been sufficiently documented. Moreover, the differential clinical profile between ischaemic stroke caused by infarctions in the territories of the ACA, middle cerebral artery (MCA) and posterior cerebral artery (PCA) is poorly defined, probably because separate analysis of ACA infarction as an individual clinical entity is rarely performed.

This single centre, retrospective study was conducted with the following aims: a) to describe the clinical characteristics and short-term outcome of stroke patients ACA territory infarction as compared with that of patients with ischaemic stroke due to MCA and PCA infarctions, and b) to identify predictors of ACA stroke.

## Methods

The database of the "Sagrat Cor Hospital of Barcelona Stroke Registry" with data of 3808 acute stroke patients was searched for those with a diagnosis of ischaemic stroke caused by occlusion in the territory of the ACA who were admitted consecutively to the Department of Neurology of the Sagrat Cor Hospital (an acute-care 350-bed teaching hospital in the city of Barcelona) between January 1986 and December 2004. Details of this on-going hospital-based stroke registry have been previously reported [4]. Data from stroke patients are entered following a standardised protocol with 161 items regarding demographics, risk factors, clinical features, laboratory and neuroimaging data, complications and outcome.

Subtypes of stroke were classified according to the Cerebrovascular Study Group of the Spanish Neurological Society [5]. These criteria has been used by our group in previous studies [4,6,7] and are consistent with those included in a recent review [8]. Definitions of cerebrovascular risk factors were those used in previous studies [4,6,7,9]. The distribution of patients in the database according to the different stroke subtypes were as follows: transient ischaemic attack (TIA) (*n *= 612), brain infarction (*n *= 2703), primary intracerebral haemorrhage (*n *= 407), spontaneous subdural hematoma (*n *= 38) and spontaneous epidural hematoma (*n *= 1). Causes of ischaemic stroke were atherothrombosis in 770 cases, cardioembolism in 763, lacunes in 733 and unusual aetiologies in 114. In 323 cases, the aetiology was unknown.

For the purpose of this study, all cases of ischaemic cerebral infarction diagnosed in 2704 patients were collected. The region of the infarction was identified on computerized tomographic (CT) scans and/or magnetic resonance imaging (MRI) studies and then topographies of the ACA (*n *= 51), MCA (*n *= 1355) and PCA (*n *= 232) were selected. These three vascular territories were defined according to previously published and validated maps of cerebral vascular territories [10] and used in other studies [4,6,7,9]. The objective of this clinical study was to assess differential features in aetiology, risk factors, clinical findings and early outcome between patients with ACA stroke and those with MCA and PCA infarctions. Prior to conducting the study, approval was obtained from the Ethical Committee of Clinical Research of the hospital.

All patients were admitted to the hospital within 48 hours of onset of symptoms. On admission, demographic characteristics; salient features of clinical and neurological examination and results of laboratory tests (blood cell count, biochemical profile, serum electrolytes, urinalysis); chest radiography; twelve-lead electrocardiography; and brain CT and/or MRI were recorded. Angio-MRI was obtained during hospitalisation in 33.3% of patients, echo-Doppler of the supra-aortic trunks in 60%, and arterial digital subtraction angiography in 9.8%. Other investigations included echocardiography in 56% of patients and lumbar puncture in 2%. Degree of clinical disability at discharge from the hospital was evaluated according to modified Rankin scale (mRS) [11], and causes of death according to the criteria of Silver et al. [12].

### Statistical analysis

Demographic characteristics, risk factors, clinical events and outcome of ACA stroke patients and those with infarctions of the MCA and PCA territories were compared using the Student's *t*-test or the Mann-Whitney *U *test for continuous variables and the chi-square (χ^2^) test (with Yate's correction when necessary) for categorical variables. Statistical significance was set at *P *< 0.05. Variables were subjected to multivariate analysis with a logistic regression procedure and forward stepwise selection if *P *< 0.10 after univariate testing. The effect of variables on the presence of infarction caused by occlusion of the ACA versus infarcts of the MCA and the PCA was studied in two multiple regression models based on demographic, vascular risk factors, and clinical and neuroimaging variables, in which the absence (codified as 0) or presence (codified as 1) of ACA infarction was the dependent variable. The level of significance was set as 0.15, and the tolerance level at 0.0001. The maximum likelihood approach was used to estimate weights of the logistic parameters. Odds ratio and 95% confidence intervals (CI) were calculated from the beta coefficients and standard errors. The hypothesis that the logistic model adequately fitted the data was tested by means of the goodness of fit χ^2 ^test. The SPSS-PC+ and the BMDP computer programmes were used for statistical analyses.

## Results

The 51 patients with ischaemic stroke caused by infarction of the ACA territory accounted for 1.3% of all cases of stroke (*n *= 3420) and 1.8% of cerebral infarction (*n *= 2407) included in the stroke registry. There were 27 men and 24 women with a mean (SD) age of 71.4 (14.7) years. Eleven patients aged 85 years or older. The following vascular risk factors in a decreasing order of frequency were observed: hypertension (54.9%), diabetes mellitus (29.4%), atrial fibrillation (23.5%) and dyslipemia (17.6%). History of previous TIA was present in 7.8% of patients. Sudden onset of neurological deficit was recorded in 96.3% of cases. Speech disturbances (dysarthria, aphasia) occurred in 43.1% of cases, sensory deficit in 29.4% and decreased level of consciousness in 7.8%. Stroke subtypes included cardioembolic infarction in 45.1% of patients, atherothrombotic infarction in 29.4%, lacunar infarct in 11.8%, infarct of unknown cause in 11.8% and infarction of unusual aetiology in 2%.

Four patients died during the hospital stay, with an in-hospital mortality rate of 7.8%. Causes of death included herniation of the brain in 1, sudden death in 1, pneumonia in 1 and septicaemia in 1. The median length of hospitalization was 12 days (25th–75th percentile, 10–24 days). Only 5 (9.8%) patients were symptom-free at the time of hospital discharge (mRS grade 0). Of the remaining 42 patients, 20 had moderate disability (mRS grade 3), 17 moderately severe disability (mRS grade 4) and 5 severe disability (mRS grade 5).

As shown in Table 1, ischaemic heart disease, atrial fibrillation, headache at the time of stroke onset, speech disturbances, altered consciousness and sensory deficit were less frequent in stroke patients with ACA infarction than in those with MCA infarction. On the other hand, headache, sensory deficit and symptom-free at discharge were less frequent, and motor deficit, speech disturbances and cardioembolism as the aetiology of stroke more frequent in patients with ACA infarction than in those with PCA infarction (Table 1).

**Table 1:** Results of univariate analysis in ischaemic stroke patients with anterior cerebral artery (ACA) infarction compared with middle cerebral artery (MCA) and posterior cerebral artery (PCA)

Data	Anterior cerebral artery	Middle cerebral artery	ACA vs MCA *P *value*	Posterior cerebral artery	ACA vs PCA *P *value^†^
Total patients	51	1355		232	

Males	27 (52.9)	635 (46.9)	0.238	128 (55.2)	0.445

Age, years, mean (SD)	74.4 (14.7)	76 (11.4)	0.331	73.9 (11.9)	0.801

Age ≥ 85 years	11 (21.6)	302 (22.3)	0.904	37 (15.9)	0.219

Cerebrovascular risk factors					

Hypertension	28 (54.9)	769 (56.8)	0.886	136 (58.6)	0.369

Diabetes mellitus	15 (29.4)	301 (22.2)	0.150	70 (30.2)	0.530

Valve heart disease	1 (2)	95 (7)	0.124	16 (6.9)	0.153

Ischaemic heart disease	4 (7.8)	223 (16.5)	0.066	36 (15.5)	0.111

Atrial fibrillation	12 (23.5)	461 (34)	0.077	62 (26.7)	0.391

Congestive heart dailure	4 (7.8)	88 (6.5)	0.431	9 (3.9)	0.190

TIA	4 (7.8)	152 (11.2)	0.314	30 (12.9)	0.225

Previous cerebral infarction	10 (19.6)	225 (16.6)	0.342	41 (17.7)	0.440

Previous cerebral haemorrhage	1 (2)	10 (0.7)	0.335	4 (1.7)	0.633

Periphery arterial disease	2 (3.9)	100 (7.4)	0.269	13 (5.6)	0.472

Obesity	4 (7.8)	51 (3.8)	0.135	11 (4.7)	0.276

Alcohol abuse (> 80 g/day)	0	39 (2.9)	0.232	1 (0.4)	0.820

Smoking (> 20 cigarettes/day)	3 (5.9)	132 (9.7)	0.261	20 (8.6)	0.376

Dyslipemia	9 (17.6)	226 (16.7)	0.458	45 (19.4)	0.474

Clinical features					

Sudden onset	27 (52.9)	722 (53.3)	0.537	108 (46.6)	0.251

Headache	0	120 (8.9)	0.010	53 (22.8)	0.000

Dizziness symptoms	2 (3.9)	14 (1)	0.112	7 (3)	0.504

Seizures	1 (2)	24 (1.8)	0.606	0	0.180

Nausea, vomiting	2 (3.9)	62 (4.6)	0.587	17 (7.3)	0.299

Speech disturbances	22 (43.1)	816 (60.2)	0.011	70 (30.2)	0.054

Altered consciousness	4 (7.8)	278 (20.5)	0.014	28 (12.1)	0.277

Motor deficit^‡^	44 (86.3)	1145 (84.5)	0.459	90 (38.8)	0.000

Sensory	15 (29.4)	554 (40.9)	0.066	119 (51.3)	0.003

Aetiological stroke subtypes			0.337		0.001

Atherothrombotic	15 (29.4)	395 (29.2)		68 (29.3)	

Lacunar	6 (11.8)	301 (22.2)		80 (34.5)	

Cardioembolic	23 (45.1)	482 (35.6)		50 (21.6)	

Unknown aetiology	6 (11.8)	124 (9.2)		20 (8.6)	

Unusual cause	1 (2)	52 (3.9)		14 (6)	

Symptom-free at discharge	5 (9.8)	149 (11)	0.507	45 (19.4)	0.072

Cardiac events	2 (3.9)	78 (5.8)	0.436	6 (2.6)	0.439

Infectious events	6 (11.8)	237 (17.5)	0.194	15 (6.5)	0.155

Respiratory events	4 (7.8)	176 (13)	0.196	12 (5.2)	0.321

Length of hospital stay, median (25th–75th percentile)	12 (10–24)	13 (9–23)	0.895	12.8 (8–22)	0.601

In-hospital deaths	4 (7.8)	235 (17.3)	0.048	9 (3.9)	0.190

Data are *n *(%) unless otherwise stated.

*P = 0.337; ^†^*P *= 0.001.

^†^Association with motor hemonegligence in 11.8%; concomitant presence of grasping in 17.6%; callosal disconnection syndrome in 5.9%.

In the multivariate analysis (Table 2), speech disturbances (odds ratio [OR] = 0.48, 95% CI 0.27–0.85) and altered consciousness (OR = 0.31, 95% CI 0.11–0.88) were independent variables of ACA stroke in comparison with MCA infarction, whereas motor deficit (OR = 9.11, 95% CI 3.8–21.8) cardioembolism as stroke mechanism (OR = 2.49, 95% CI 1.21–5.14) and sensory deficit (OR = 0.35, 95% CI 0.17–074) were independent variables associated with ACA stroke as compared with PCA infarction.

**Table 2:** Variables associated with ischaemic stroke caused by anterior cerebral artery (ACA) infarction

Variable	β	SE (β)	Odds ratio (95% CI)	*p*
ACA *versus *MCA infarctions				

Model based on demographics, vascular risk factors and clinical variables*				

Speech disturbances (dysarthria, aphasia)	-0.728	0.289	0.48 (0.27–0.85)	0.012

Altered consciousness	-1.159	0.526	0.31 (0.11–0.88)	0.028

ACA *versus *PCA infarctions				

Model based on demographics, vascular risk factors, clinical features and topographic and aetiological variables^†^				

Motor deficit	2.210	0.445	9.11 (3.8–21.8)	0.000

Cardioembolism	0.914	0.269	2.49 (1.21–5.14)	0.013

Sensory deficit	-1.036	0.377	0.35 (0.17–0.74)	0.006

*^β ^= -2.748; SE (β) = 0.197; goodness-of-fit χ^2 ^= 0.271; *df *= 2; *p *= 0.873; area under the ROC curve = 0.628; sensitivity 51%; specificity 69%; positive predictive value 6%; negative predictive value 97%; correct classification 68.8%.

^†β ^= -2.739; SE (β) = 0.436; goodness-of-fit χ^2 ^= 7.967; *df *= 5; *p *= 0.158; area under the ROC curve = 0.811; sensitivity 65%; specificity 75%; positive predictive value 38%; negative predictive value 90%; correct classification 72.9%.

## Discussion

Data regarding the frequency of cerebral infarctions in the territory of the ACA in the different stroke registries are scarce, the number of which varies between 27 in the Lausanne Stroke Registry [13] and 47 in the Ege Stroke Registry [14]. The clinical series of 51 patients here described is the largest group so far reported in the literature (Table 3). The present results indicate that infarctions in the ACA territory is a subgroup of unusual cerebral infarcts, accounting for 1.8% of all cases of stroke and 1.3% of all cases of cerebral infarctions. This percentage is similar to 1.9% observed in the series of Moulin et al. [15], slightly higher than 1.1% in the series of Vemmos et al. [16] and 1.3% of Kumral et al. [13] but lower than 2.3% in the clinical series of Gandehari and Izadi [17].

**Table 3:** Cerebral infarcts in the territory of the anterior cerebral artery (ACA). Main series reported in the literature

First author, year [reference]	Patients	Clinical series	Frequency cardioembolic aetiology (%)	Frequency total infarctions (%)	Frequency total Stroke (%)	In-hospital mortality (%)
Bogousslavski, 1990 [13]	27	Lausanne Stroke Registry (*n *= 1490)	26	Not stated	1.8	Not stated

Moulin, 2000 [15]	35	Besançon Stroke Registry (*n *= 1776)	45.7	1.9	Not stated	14.3

Vemmos, 2000 [16]	10	Athens Stroke Registry (n = 1042)	50	1.1	0.9	Not stated

Kumral, 2002 [14]	48	Ege Stroke Registry (*n *= 4750)	27	1.3	1	0

Gandehari, 2007 [17]	32	Khorasan Stroke Registry (*n *= 1392)	Not stated	2.3	Not stated	Not stated

Present series, 2009	51	Hospital Sagrat Cor Stroke Registry (*n *= 3808)	45.1	1.8	1.3	7.8

It should be noted that 21.6% of our patients were older than 85 years of age. This finding is consisted with results of other studies [18-20] and highlights the increasing prevalence and clinical relevance of first-ever stroke in the oldest old population segment of developed countries.

The aetiology of ACA infarctions is poorly defined, although in our study, the most frequent aetiopathogenic mechanism was cardioembolism in 45% of cases, followed by atherothrombosis in 29% and lacunes in 12%. In 12% of the cases, the cause was unknown. Infarctions were considered of cardioembolic origin according to criteria recommended by the Cerebrovascular Study Group of the Spanish Society of Neurology [5] and defined as a medium-to-large size cerebral infarction, usually of cortical topography in which in the absence of other etiologies, some of the following emboligenous cardiac disorders are documented: atrial flutter or atrial fibrillation, intracardiac thrombus or tumor, rheumatic valve disease, mitral or aortic valve prosthesis, endocarditis, sinus node disease, left ventricular aneurysm after acute myocardial infarction, acute myocardial infarction in the acute phase (less than 3 months) or global cardiac hypokinesia. The identification of cardioembolism as the aetiology of stroke in 45% of our patients is similar to the percentages of 45.7% reported in the studies of Moulin et al. [15] and Vemmos et al. [50%] but clearly higher than 25% found in the studies of Bougousslavki and Regli [13] and 27% in the study of Kumral et al. [14]. However, all these percentages are consistently higher than the frequency of about 20% attributed to cardioembolism when cerebral infarctions are analysed as a whole [21,22]. In this respect, this aetiological aspect is important because a diagnosis of cardioembolism has practical implications in the management of these patients. In the presence of a cerebral infarction in the ACA territory, the cardioembolic mechanism should be considered and, therefore, the possibility to indicate early anticoagulation at therapeutic doses as a secondary prevention of cardioembolic stroke [8].

Patients with infarcts in the ACA territory have a relatively favourable short-term prognosis as shown by an in-hospital mortality rate of 7.8%, clearly lower that 17.3% of MCA infarctions. Other authors have reported mortality rates between 0% in the series of Kumral et al. [14] and 14.3% in the study of Moulin et al. [15].

Neuroimaging data (brain CT and/or MRI) was indispensable to confirm the vascular topography of the lesion and to classify patients into the three groups of infarcts in the territory of the ACA, MCA and PCA. The remaining diagnostic studies (laboratory data, echo-Doppler of the supra-aortic trunks, arterial digital subtraction angiography, etc.) were useful to classify patients into the different ischemic stroke subtypes.

It should be noted that ACA infarcts present a clinical profile clearly different than the remaining cerebral hemispheric infarctions. Speech disturbances and altered consciousness were independent variables associated with ACA infarctions as compared with MCA stroke. The lower frequency of dysarthria or aphasia is explained because both Broca's motor speech area and sensory speech area of Wernicke are located in the vascular territory of the MCA [1,10]. The lower frequency of decreased consciousness may be related to the small diameter of the lesion in ACA in comparison with MCA infarctions. A larger volume of brain ischaemia increases the risk of intracranial hypertension and consequently more frequent impairment of the level of vigilance at the beginning of the focal neurological deficit [1].

Variables associated with ACA stroke versus infarctions in the vascular territory of the PCA were motor deficit, cardioembolism and sensory deficit. Motor deficit with a characteristic crural distribution is the most common neurological sign of ACA stroke and was present in 86.3% of our patients as compared with a prevalence of 93.3% in the series of Kumral et al. [14] and 96% in the series of the Lausanne Stroke Registry [13]. Infarctions in the ACA territory usually involve the paracentral component of the frontal lobe affecting motor neurons with a somotatotopic distribution mostly related to the lower extremities [1]. Cardioembolism is the most frequent aetiology of ACA infarction as compared with infarcts in the PCA territory in which cardioembolism was the third cause after lacunar infarctions and atherothrombosis. On the other hand, the lower frequency of sensory deficit in ACA stroke than in PCA infarction is explained because the deep vascular territory of the PCA includes the ventroposterolateral thalamic nucleus [23].

## Conclusion

Cerebral infarcts in the ACA territory are infrequent and account for only 1.3% of all cases of cerebral infarction and 1.3% of all cases of stroke. Cardioembolism is the main aetiology of ACA stroke. Patients with ACA infraction have a favourable short-term prognosis and show a clinical profile different than the remaining cerebral hemispheric infarcts.

## Abbreviations

ACA: anterior cerebral artery; CI: Confidence interval; CT: Computed tomography; MCA: middle cerebral artery; MRI: Magnetic resonance imaging; OR: Odds ratio; PCA: Posterior cerebral artery; ROC: Receiver operating characteristics; SD: standard deviation; TIA: Transient ischaemic attack.

## Competing interests

The authors declare that they have no competing interests.

## Authors' contributions

AA was the principal investigator, designed the study, diagnosed and took care of the patients, contributed to analyze the data, interpreted the results, wrote the paper, and prepared the final draft. He was also responsible for editorial decisions including the selection of the journal. LG-E was the statistician, participated in the study design, analysis and interpretation of data, and wrote the part of the paper related to the statistical analysis. NS, AR, MO and JM participated in the collection of data medical care of the patients, analysis of results, and review of the manuscript for intellectual content. All authors read and approved the final manuscript.

## Pre-publication history

The pre-publication history for this paper can be accessed here:


